# Knowledge mapping of immunotherapy for thyroid cancer from 1980 to 2022: A review

**DOI:** 10.1097/MD.0000000000035506

**Published:** 2023-09-29

**Authors:** Ran Ding, Hongguan Jiao, Yuanlin Piao, Weiyi Tian

**Affiliations:** a School of Health Preservation of Traditional Chinese Medicine, Guizhou University of Traditional Chinese Medicine, Guiyang, People’s Republic of China; b School of Information Engineering, Guizhou University of Traditional Chinese Medicine, Guiyang, 550025 People’s Republic of China; c Virginia University of Integrative Medicine, Vienna, VA; d School of Basic Medicine, Guizhou University of Traditional Chinese Medicine, Guiyang, People’s Republic of China.

**Keywords:** bibliometrics, co-occurrence, immunotherapy, thyroid cancer, visualization

## Abstract

With the gradual development of immunotherapy for thyroid cancer, relevant research has increased. To better understand the current situation, development trend, evolution process, and research hotspots of this field, we conducted this comprehensive bibliometrics visual analysis. We retrieved papers published from 1980 to 2022 from Web of Science Core Collection on January 31, 2023. CiteSpace, Pajek, VOSviewer, R-Bibliometrix, and Scimago Graphics are the tools to perform the analysis. Analysis methods mainly include co-occurrence analysis and cluster analysis. Analysis objects are countries or regions, institutions, authors, journals, and keywords, etc. In terms of publication number, the recent decade has witnessed rapid growth. USA was the most prolific country and has the most influence in the cooperation team. Sweden took the lead in focus on this research field and lasted for 21 years. Garden State Cancer Center was released most papers (28). INSERM played a major role in institutional cooperation. Goldenberg DM published the most papers (48), with H-Index 25 and G-Index 43. *Journal of Nuclear Medicine* has the greatest papers published (41). The average impactor factor of the top 10 journals is 7.2058. The top keywords with high burst strength are: radioimmunotherapy (14.85), monoclonal antibody (13.78), non hodgkins lymphoma (12.54). The research field of immunotherapy for thyroid cancer will be further developed. This study provides a valuable reference for future research in the field.

## 1. Introduction

Thyroid cancer (TC) is a very common endocrine malignant tumor, unlike many other malignant tumors, TC has the unique characteristic of being able to affect individuals of all age groups, including children, with the median age at diagnosis being 51 years, approximately 43% of new cases occur in individuals aged between 45 and 64 years. TC often presents with neck nodules, lumps, swelling, pain, and swallowing, dyspnea, cough and other symptoms. There are 3 most common histologic subtypes of thyroid cancer, that is, papillary thyroid cancer, follicular thyroid cancer, and Hurthle cell cancer. Papillary thyroid cancer is the most common type, accounting for about 90% of new cases and the prognosis is good. Depending on a survey, the morbidity of TC has tripled in the last 30 years.^[1]^ However, due to its slow growth and hidden symptoms. Many cases were not found during the whole life until autopsy.^[2–5]^ A small number of thyroid nodules were detected during physical examination. Most thyroid nodules can be identified by ultrasonography.^[6]^ Thyroid scans are commonly performed to evaluate thyroid cancer.^[7–9]^ Technologies like computed tomography, magnetic resonance imaging and fine needle aspiration cytology are often used to diagnose TC.^[10]^ At present, whether there are suitable biomarkers to diagnose TC is still controversial.^[11]^ There are many therapies to intervene in TC, such as thyroidectomy and lymph node dissection when the disease is limited to local structures. For different TC, radioactive iodine is used for treating radiosensitive TC.^[12]^ For metastatic TC, Adriamycin is used in cytotoxic chemotherapy.^[13]^ Targeted agents are part of the current standard treatment methods for TC when it has spread beyond the thyroid gland (metastatic disease) and cannot be effectively treated with surgery or radioactive iodine therapy.^[14]^ Immunotherapy is usually treating allergic diseases, such as allergic rhinitis, allergic asthma and other allergic diseases.^[15–17]^ Since approval of immune checkpoint inhibitors as the main immunotherapy for cancers by Food and Drug Administration of USA in 2011, it has become one of the pillar therapies for cancer treatment.^[18]^ Immunotherapy is gradually used for the treatment of various cancers.^[19–21]^ It has also been widely applied in TC.^[12,22–26]^ With the wide application of immunotherapy in TC, a large number of related studies have emerged, accompanied by the publication of scientific papers. Bibliometrics is the application of mathematical and statistical methods to analyze data in relevant research fields. Some bibliometric analyses have analyzed immunotherapy for different cancers,^[27–30]^ but as far as we know, there is no bibliometric analysis of immunotherapy for TC. In order to clearly understand the development status of this field, this study applies the bibliometric method to visualize and analyze the relevant papers of immunotherapy for TC.

## 2. Materials and methods

**Figure 1.**
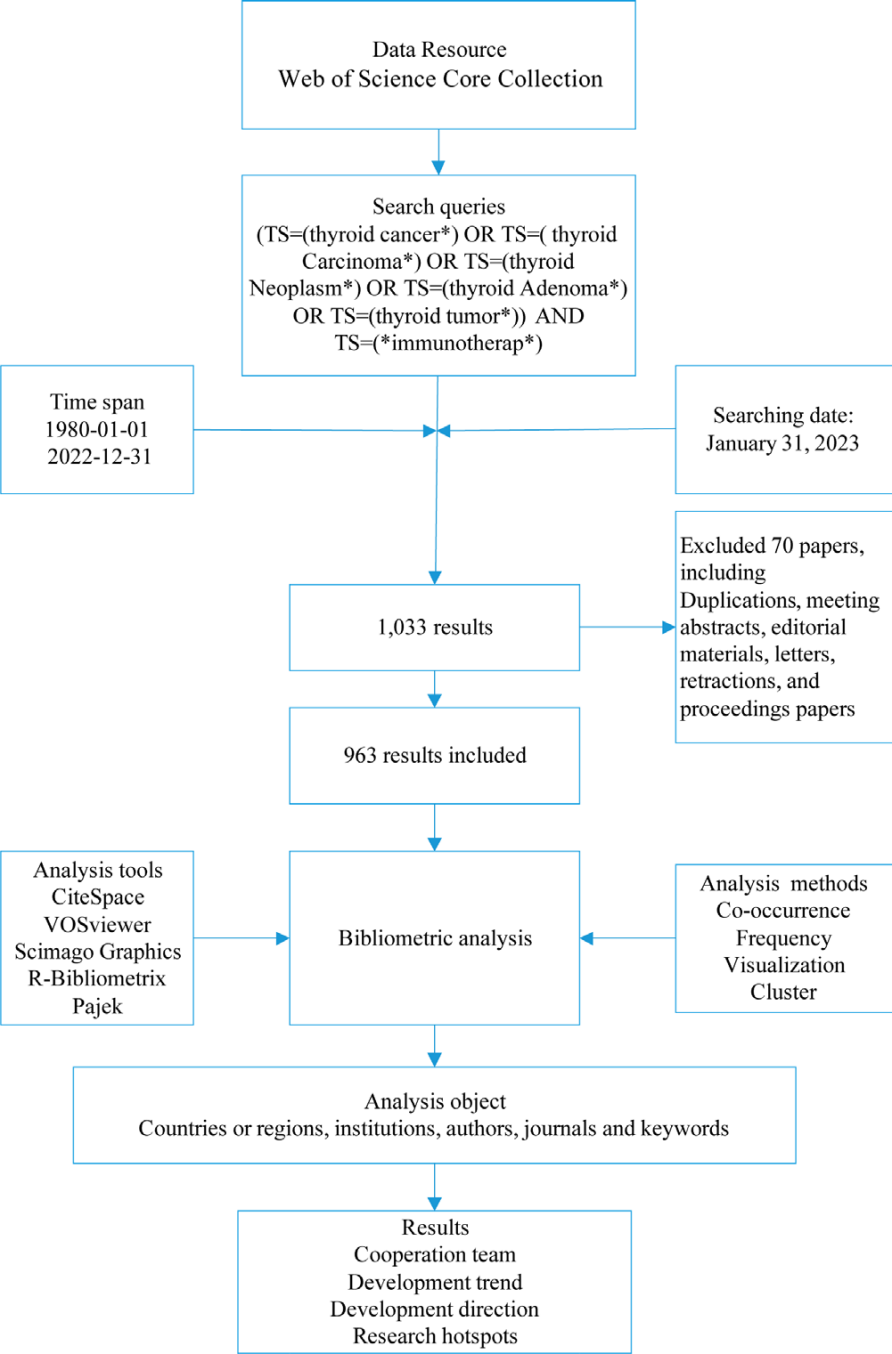
Flowchart of this study.

Figure 1 shows the flow chart of this study. First, search and download relevant paper data from the database. The visualization software programs of bibliometrics are used for co-occurrence analysis, frequency analysis and cluster analysis to analyze the relevant contents of countries or regions, institutions, journals, keywords, etc. The purpose of the research is to obtain the overview, development trend, cooperation team, and research hotspot of the research field.

### 2.1. Data collection

web of science core collection (WoSCC) was selected as the data source. The search queries were: [TS = (thyroid cancer*) OR TS = (thyroid Carcinoma*) OR TS = (thyroid Neoplasm*) OR TS = (thyroid Adenoma*) OR TS = (thyroid tumor*)] AND TS=(*immunotherap*), Time span = January 01, 1980 to December 31, 2022. Searching date: January 01, 2023. A total of 1033 results were obtained. Since the data included in the article does not contain any personal information of patients, ethical approval is not required.

### 2.2. Analysis methods and tools

We used descriptive and co-occurrence analysis methods to analyze the obtained data.^[31,32]^ CiteSpace (6.1.R6), Pajek (64 5.16), VOSviewer (1.6.17), and Scimago Graphics (Beta 1.0.26) were used as the analyzing tools to perform the visual analysis.^[33–41]^ These software programs are based on co-occurrence analysis and cluster analysis to analyze the cooperation relationship between items such as countries or regions, institutions, authors and the cluster of keywords.^[34,42]^ In general, the size of items (nodes) in the map indicates the value of the item, such as the number of published papers or citation. The number and thickness of links between nodes shows the relationship between them. Centrality shows the importance of a member of a cooperative team. Burst indicates an item has received more attention over a period of time.^[32,43–46]^ R-Bibliometrix was utilized to analyze the thematic evolution and trend.^[47,48]^

## 3. Results

### 3.1. Publication output analysis

**Figure 2.**
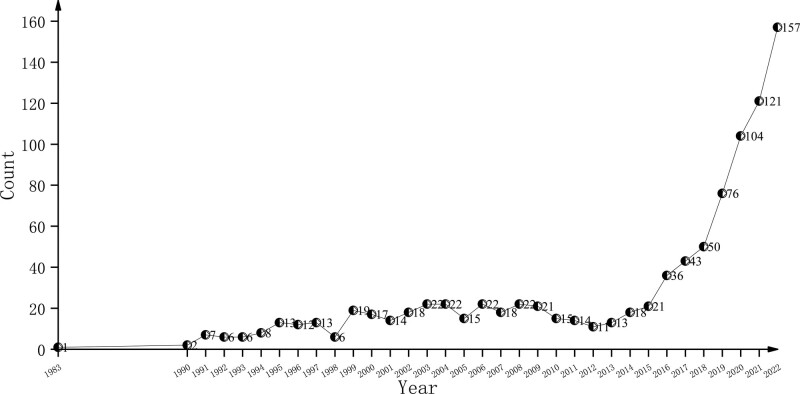
The annual output of publication on immunotherapy for thyroid cancer.

In all 963 articles, the first article appeared in 1983.^[49]^ Figure 2 shows the trend of publishing volume changes by year. The period from 1984 to 1989 showed that there was not any relevant paper published. From 2 papers in 1990 to 13 papers in 1997, it is a slow growth process. From 1999 to 2009, it was a plateau period, with an annual volume of about 20 papers. There was a slight decline in the next 3 years after 2010. Then the number of papers increased rapidly from 13 in 2013 to 157 in 2022. In the last 10 years, it accounted for 66% of the total number of papers published.

### 3.2. Analysis of countries or regions

**Figure 3.**
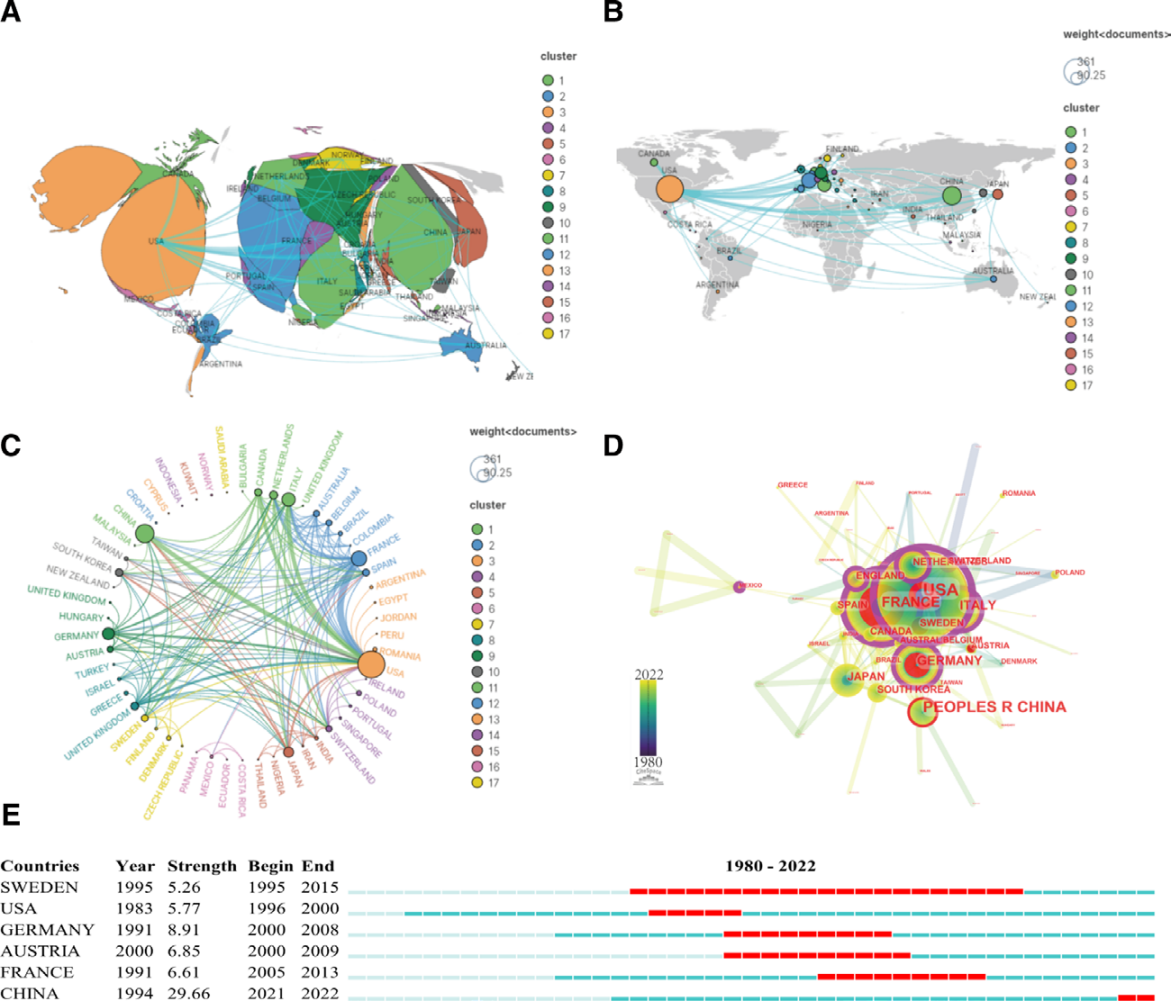
Map of national or regional cooperation. (A) Map of countries or regions according to the proportion of published papers. (B) World distribution map of national or regional cooperation. (C) Circular chart of national or regional cooperation. (D) The map of cooperation relationship of countries or regions over time. (E) Top 6 Countries with the strongest bursts strength.

A total of 963 papers have been produced in 56 countries or regions. Table 1 shows the top 10 countries in terms of the publication number. USA is the 1 with the highest volume of documents (365). It is greater than twice that of China (168), which ranks second. Figure 3 shows the map of national or regional cooperation. Each node represents a country or region. The size of each node represents the number of published papers. The number and thickness of links between nodes indicate the strength of their cooperation relationship. Figure 3A shows the map of countries or regions according to the proportion of published papers. Different colors represent different clusters, and countries or regions with the same color represent closer cooperation. The size of the area of each country or region indicates the number of published papers in this research field. Figure 3B shows the world distribution map of national or regional cooperation. The research in this field is distributed in all continents, but mainly concentrated in economically developed countries or regions. Figure 3C shows the cooperation relationship between countries in a circular map. Countries or regions with close cooperation constitute a cluster in the same color. Those that do not constitute a cooperative relationship with other countries or regions form their own clusters. There are 17 clusters in total, and 10 clusters are relatively large and representative, clusters representing different cooperation teams. The first cluster is green, its main members are Italy, Netherlands, Canada; The second cluster is blue, its members are France, Spain, Australia, Belgium, Brazil; The third cluster is orange, its members are USA, Romania, Argentina; The 4th cluster is purple, its members are Switzerland, Poland, Portugal, Singapore; The fifth cluster is dark orange, its members are Japan, India, Iran. The nodes in Figure 3D show the change of the country or region from the inside to the outside, transition from dark blue to green and then yellow, and the time from far to near. Each ring represents a year. The purple circle around the node indicates that the centrality of the country or region is relatively strong (Table 1). Top prolific countries or regions with high centrality are USA (0.45), France (0.24), Italy (0.16), Germany (0.14), and England (0.14). They play an important connecting role in the cooperative team. The red ring in the node indicates that the burst value of the node is relatively large, and the corresponding ring position indicates the period with high burst value (Fig. 3E). This indicates that they began to pay great attention to this research field at a certain period. Among them, Sweden, USA, Germany, Austria and France began to draw attention to this field relatively early (from 1995 to 2005). China began to focus attention on this research relatively late (2021). However, China has the largest burst strength (29.66), indicating that China has recently invested more in this research field.

**Table 1:** Ranking of Top 10 countries or regions on immunotherapy for thyroid cancer.

Ranking	Countries or Regions	Record Count	% of 963	Centrality	Year
1	USA	365	37.902	0.45	1983
2	China	168	17.445	0.07	1994
3	France	114	11.838	0.24	1991
4	Italy	89	9.242	0.16	1990
5	Germany	71	7.373	0.14	1991
6	Japan	51	5.296	0.1	1992
7	Netherlands	32	3.323	0.12	1999
8	England	29	3.011	0.14	1994
9	South Korea	28	2.908	0.08	2007
10	Canada	26	2.7	0.04	1994

### 3.3. Analysis of institutions

**Figure 4.**
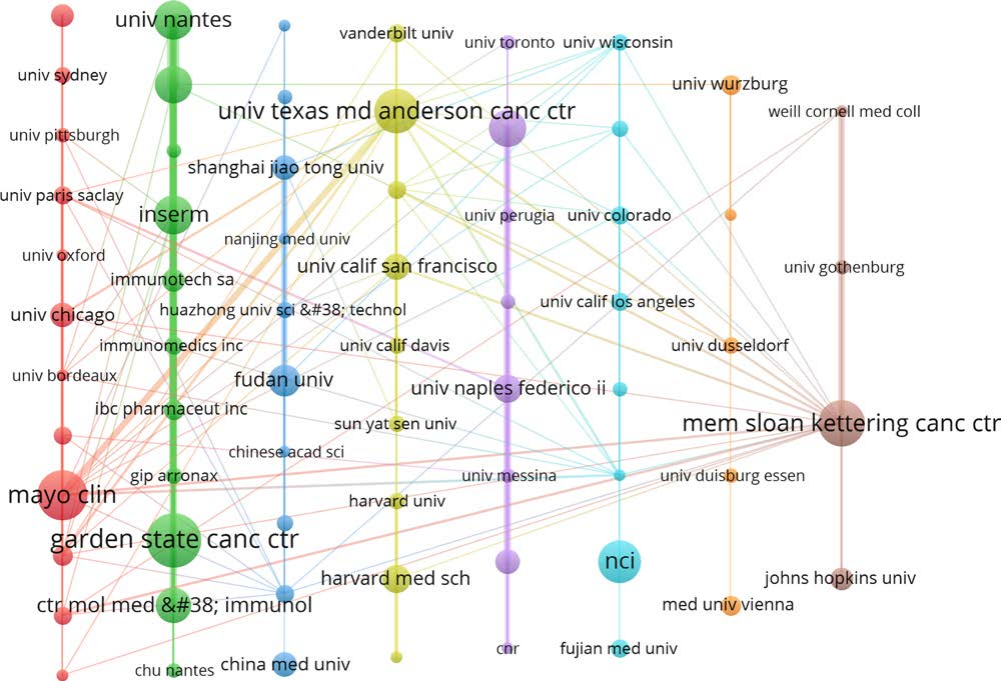
Institution cluster vertical distribution map on immunotherapy for thyroid cancer.

A total of 1283 institutions participated in writing of these 963 papers. Table 2 displays the top 10 institutions in terms of publication number, the top 10 institutions published 22.74% of the total. Six of them come from the USA, 2 from France, and the other 2 from Italy and China. Mayo Clin and Univ Texas MD Anderson Canc Ctr have greater academic influence. The average number of citations per paper is 82 and 85 respectively. From the perspective of centrality, the values of INSERM (0.11) and Memorial Sloan Kettering Cancer Center and (0.07) are the highest, indicating that they occupy an important position in the cooperation team. Sixty-seven institutions with cooperative relationships that have published more than or equal to 5 papers were incorporated into the cluster map (Fig. 4). There are 8 clusters in total. Each cluster represents a cooperative team, indicating that their cooperation between the members of the same cluster is relatively close. The first cluster includes institutions sort by frequency: Mayo Clin, Univ Chicago, Wayne State Univ, Inst Gustave Roussy, Gustave Roussy, Stanford Univ, Univ Paris Saclay, Univ Sydney, Univ Pittsburgh, Albert Einstein Coll Med, Univ Bordeaux, Univ Oxford; The second cluster includes: Garden State Canc Ctr, INSERM, Univ Nantes, Univ Hosp, Ctr Mol Med & Immunol, Immunotech Sa, Ibc Pharmaceut Inc, Immunomedics Inc, Gip Arronax, Chu Nantes, Inst Biol; The third cluster includes: Fudan Univ, China Med Univ, Shanghai Jiao Tong Univ, Chinese Acad Med Sci, Chinese Acad Med Sci and Peking Union Med Coll, Huazhong Univ Sci & Technol, Zhejiang Univ, Chinese Acad Sci, Nanjing Med Univ, Zhengzhou Univ; The 4th cluster includes: Univ Texas MD Anderson Canc Ctr, Harvard Med Sch, Univ Calif San Francisco, Univ Michigan, Vanderbilt Univ, Harvard Univ, Sun Yat Sen Univ, Univ Calif Davis, Brigham and Womens Hosp; The 5th cluster includes: Univ Pisa, Univ Naples Federico Ii, Sapienza Univ Rome, Univ Messina, Univ Padua, Univ Perugia, Univ Toronto, CNR.

**Table 2:** Top 10 institutions on Immunotherapy for Thyroid Cancer.

Ranking	Institutions	Institution Abbreviations	Country	Documents	Citations	Average Citations	% of 963	Centrality	Year
1	Garden State Cancer Center	Garden State Canc Ctr	USA	28	1210	43	2.91	0.03	1999
2	Mayo Clinic	Mayo Clin	USA	24	1961	82	2.49	0.04	2006
3	Memorial Sloan Kettering Cancer Center	Mem Sloan Kettering Canc Ctr	USA	22	1216	55	2.28	0.07	1999
4	National Cancer Institute	NCI	USA	21	1070	51	2.18	0.02	1991
5	University of Texas MD Anderson Cancer Center	Univ Texas Md Anderson Canc Ctr	USA	21	1780	85	2.18	0.1	2017
6	National Institute of Health and Medical Research	INSERM	France	19	970	51	1.97	0.11	1999
7	University of Nantes	Univ Nantes	France	19	719	38	1.97	0.01	2005
8	University of Pisa	Univ Pisa	Italy	18	593	33	1.87	0.01	2010
9	Center for Molecular Medicine and Immunology	Ctr Mol Med and Immunol	USA	17	653	38	1.77	0.01	1997
10	Fudan University	Fudan Univ	China	15	164	11	1.56	0.01	2004

### 3.4. Analysis of authors

A total of 5280 authors participated in the creation of 963 papers. Table 3 lists the top 10 authors in the number of publishing articles. Indicators such as h-index, g-index and m-index are listed respectively. These indicators are calculated on the basis of the number of publications and the number of citations to evaluate the influence of an author, institution or journal.^[50–54]^ In terms of the number and influence of published papers, the top 6 authors are all ranked high. Bodet-milin C, ranked 10th in the number of published papers, started late (2006). Although the number of papers is not large (12), the M-Index is high (0.5), which indicates that this author has great impact.

**Table 3:** Top Ten Authors on Immunotherapy for Thyroid Cancer.

Ranking	Authors	Count	% of 963	H-index	G-index	M-index	Citations	Start year
1	Goldenberg DM	48	4.984	25	43	0.862	1876	1995
2	Barbet J	44	4.569	21	41	0.724	1722	1995
3	Kraeber-bodere F	38	3.946	18	35	0.72	1241	1999
4	Chatal JF	36	3.738	20	36	0.69	1723	1995
5	Sharkey RM	27	2.804	20	27	0.69	1439	1995
6	Faivre-chauvet A	23	2.388	15	23	0.6	697	1999
7	Rousseau C	14	1.454	8	14	0.333	371	2000
8	Schott M	14	1.454	7	14	0.292	263	2000
9	Vuillez JP	14	1.454	8	14	0.308	643	1998
10	Bodet-milin C	12	1.246	9	12	0.5	318	2006

**Figure 5.**
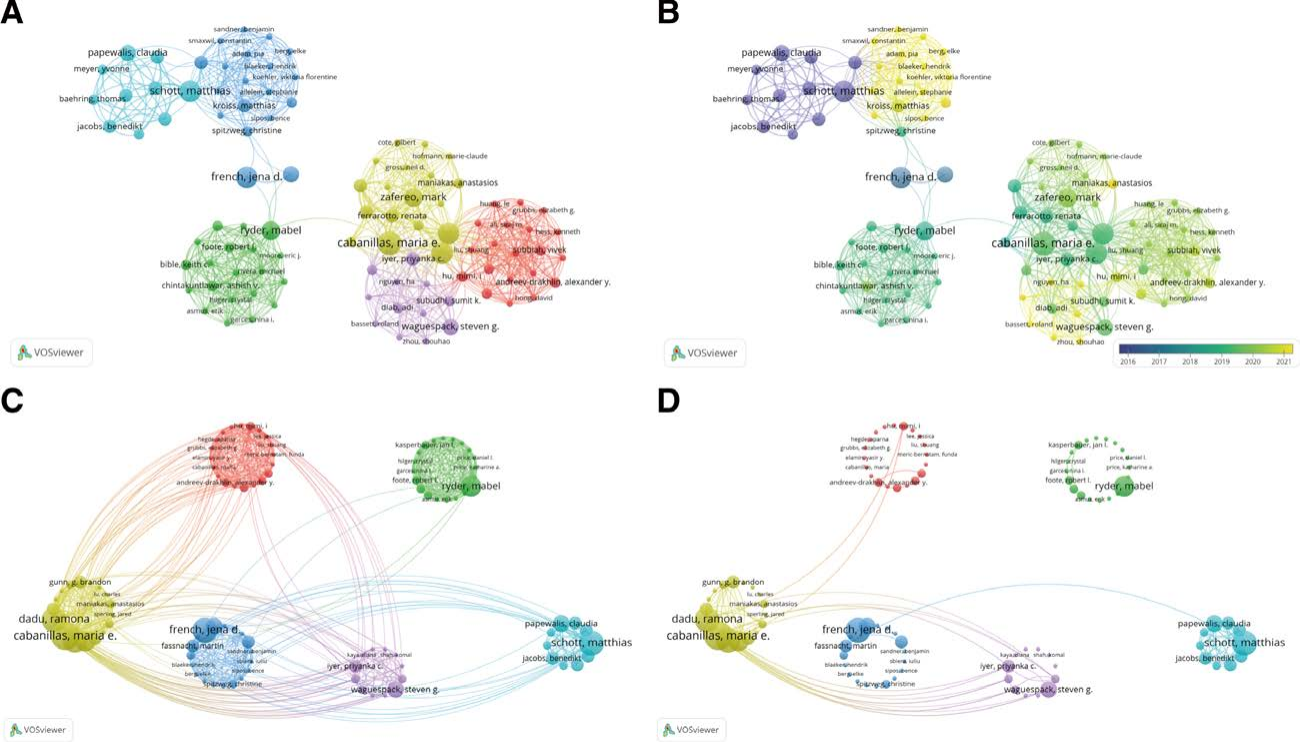
Author cooperation network map on immunotherapy for thyroid cancer. (A) The author’s cooperative relationship. (B) The author’s cooperative relationship changes over time. (C) The author’s cooperative relationship expanded map (links ≥ 1). (D) The author’s cooperative relationship expanded map (links ≥ 2).

Figure 5 displays the author’s clustering map. It shows the author’s cooperation. A total of 99 authors who have published more than 2 articles and have cooperative relations are illustrated in the figure. There are 6 clusters. Different colors represent different cooperation teams (Fig. 5A). Figure 5B shows the cooperation happened between different authors or teams timely. The change of time from far to near is purple, green, and yellow respectively. Figure 5C and Figure 5D are expanded maps of the teams. From the comparison of the 2 figures (Figure 5C and Figure 5D), the links between the authors are not strong. It shows that mature cooperation between authors in this field has not yet been formed.

### 3.5. Analysis of journals

A total of 384 journals have included the 963 papers. Table 4 displays the top 10 journals with the highest productivity. The papers printed in the top 10 journals account for 22.74% of all papers. *Journal of Nuclear Medicine* has the greatest papers published, with 41 articles. It holds the highest H-Index (25) and G-Index (41). Clinical Cancer Research has the highest impactor factor (IF) 11.082. The average IF of the top 10 journals is 7.2058. In terms of SCI subject category, The *Journal of Nuclear Medicine belongs to Radiology, Nuclear Medicine and Medical Imaging category*; 4 of the top *Journals are in Endocrinology and Metabolism category*; 3 of them are in *Oncology category*; The categories of the other 2 *Journals are Immunology and Medicine, Research and Experimental*. From the position of journals in SCI subject category, 2 journals rank in the top 10% (2.94%, 6.94%) of the category; 5 in the top 25%; 2 ranking around 30%; this shows that the research field is relatively influential in the industry.

**Table 4:** Top 10 Journals Publishing Papers on Immunotherapy for Thyroid Cancer.

Ranking	Publication Titles	Count	% of 963	H-index	G-index	Times CITED	IF*	SCI subject category	Position	% of Position
1	*Journal of Nuclear Medicine*	41	4.258	25	41	1878	11.082	Radiology, Nuclear Medicine and Medical Imaging	4/136	2.94
2	*Thyroid*	27	2.804	16	27	889	6.506	Endocrinology and Metabolism	28/146	19.18
3	*Cancers*	23	2.388	9	14	207	6.575	Oncology	60/245	24.49
4	*Cancer Biotherapy and Radiopharmaceuticals*	22	2.285	9	19	383	3.632	Medicine, Research and Experimental	84/139	60.43
5	*Frontiers In Oncology*	21	2.181	5	9	106	5.738	Oncology	78/245	31.84
6	*Journal of Clinical Endocrinology and Metabolism*	20	2.077	14	20	1236	6.134	Endocrinology and Metabolism	32/146	21.92
7	*Clinical Cancer Research*	18	1.869	14	18	850	13.801	Oncology	17/245	6.94
8	*Cancer Immunology Immunotherapy*	17	1.765	11	17	447	6.630	Immunology	49/162	30.25
9	*Endocrine-Related Cancer*	15	1.558	11	15	526	5.905	Endocrinology and Metabolism	35/146	23.97
10	*Frontiers in Endocrinology*	15	1.558	5	13	182	6.055	Endocrinology and Metabolism	33/146	22.60

IF = impactor factor.

* IF according to Journal Citation Reports (2021).

### 3.6. Analysis of keywords

**Figure 6.**
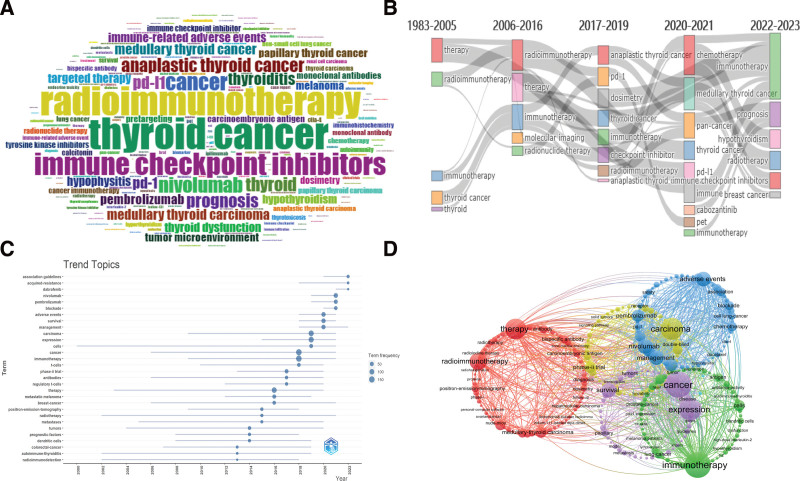
Keyword analysis. (A) Word cloud of the top 200 keywords on immunotherapy for thyroid cancer. (B) Thematic evolution of five time slices on immunotherapy for thyroid cancer. (C) The top 30 keywords of the latest trend topics on immunotherapy for thyroid cancer. (D) Keywords clustering map on immunotherapy for thyroid cancer.

A total of 4121 keywords were extracted from 963 papers, including 2358 Keywords Plus and 1763 author’s keywords. Figure 6A shows the word cloud of the top 200 Keywords. The size of the font indicates the frequency. The top 10 keywords are: immunotherapy (232), thyroid cancer (76), radioimmunotherapy (70), immune checkpoint inhibitors (54), cancer (41), nivolumab (37), anaplastic thyroid cancer (33), prognosis (33). Figure 6B shows the thematic evolution map. Each column represents a time slice. With the change of time, different keywords appear, which represent the emergence of new topics. From 1983 to 2005, the time slice’s immunotherapy, radioimmunotherapy, and thyroid cancer was a preliminary development process from the concept. By 2006 to 2016, the molecular imaging, and 2017 to 2019, anaplastic thyroid cancer, anaplastic thyroid carcinoma, checkpoint inhibitor, pd-1 (programmeddeath-1), are the emergence of the diagnosis, classification and specific treatment of the disease. The 2020 to 2021 and 2022 to 2023 phases represent the recent changes. There are some new classification and treatment plans, and the proposal of methods, such as cabozantinib, chemotherapy, immune checkpoint inhibitors, hypothyroidism, medullary-thyroid cancer, and other diseases related to TC, such as breast-cancer and pan-cancer. Figure 6C shows the latest trend topics. The research hotspots in the last 5 years include t-cells, carcinoma, expression, cells, adverse events, survival, management, nivolumab, pembrolizumab, blockade, association guidelines, acquired-resistance, dabrafenib. Figure 6D shows the keywords cluster map. The keywords with frequency greater than or equal to 5 constitute 5 large clusters. Each cluster represents different research directions and is represented by different colors. The largest cluster has 73 members. There are 21 keywords whose frequency is greater than or equal to 15. They are therapy, radioimmunotherapy, medullary-thyroid carcinoma, non hodgkins-lymphoma, monoclonal antibody, antibody, bispecific antibody, monoclonal antibodies, positron-emission-tomography, diagnosis, carcinoembryonic antigen, dosimetry, medullary-thyroid cancer, radiotherapy, bivalent hapten, metastases, thyroid cancer, trial, phase-i trial, radioiodine therapy, receptor radionuclide therapy. The main members of the second cluster are: immunotherapy, cells, t-cells, lung-cancer, antigen, regulatory t-cells, lymphocytes, dendritic cells, prostate-cancer, antitumor immunity, in vitro, tumor, hypothyroidism, in vivo, melanoma patients, thyroid-dysfunction. The third includes nivolumab, adverse events, management, ipilimumab, pembrolizumab, metastatic melanoma, melanoma, open-label, chemotherapy, blockade, association, advanced melanoma, antibodies, cell lung-cancer, cancer-immunotherapy, pd-1, safety, receptor, ipilimumab-induced hypophysitis, squamous-cell carcinoma, docetaxel. The 4th cluster includes carcinoma, phase-ii trial, efficacy, double-blind, phase-ii, prognostic-factors, association guidelines, braf, combination, multicenter, mutations, radioactive iodine. The fifth cluster includes cancer, expression, survival, breast-cancer, tumors, disease, papillary, gene-expression, pd-l1, risk, tumor-associated macrophages, guidelines, autoimmunity.

### 3.7. Analysis of burst keywords

**Figure 7.**
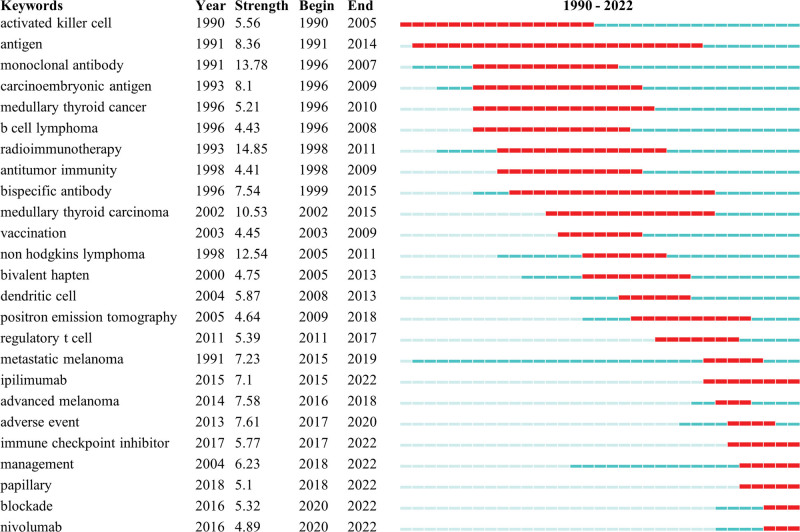
Top 25 keywords with the strongest bursts.

Burst keyword indicates that a topic has received more attention in a certain period of time.^[55]^ Figure 7 displays the top 25 keywords with the strongest bursts. The first burst keyword appeared in 1990 was the activated killer cell, which lasted 16 years until 2005. In terms of burst strength, the top 3 are: radioimmunotherapy (14.85), monoclonal antibody (13.78), non hodgkins lymphoma (12.54). The keywords that have received more attention in the past 2 decades include bivalent hapten, dendritic cell, positron-emission tomography, regulatory t cell, metastatic melanoma, ipilimumab, advanced melanoma, adverse event, immune checkpoint inhibitor, management, papillary, blockade, nivolumab.

### 3.8. Analysis of citation

All 963 papers were cited 26,991 time. The average citations per paper was 28.03. The h-index was 82. The top 10 highly cited articles are presented in Table 5. Six of the top 10 articles were published after 2015, indicating that research in this field has made rapid progress and that very new articles can receive more attention in a short period of time. These highly cited papers have confirmed the efficacy of immunotherapy, and also discussed the side effects or adverse effects of immunotherapy. The mechanism and safety of immunotherapy are the main topics that scholars take an interest in. The article with the highest number of citations was published by Marabelle, Aurelien et al in 2020.^[34]^ They performed a retrospective study of pembrolizumab monotherapy in 1066 patients with 10 types of unresectable or metastatic tumors, including thyroid cancer. They concluded that high tissue tumor mutational burden may be a new and useful predictive biomarker for the response to the therapy. The second article is a review of the adverse events profile of anti-programmed cell death-1 or anti cytotoxic-T-lymphocyte-associated antigen 4 monoclonal antibodies.^[56]^ The third high cited article believed that the treatment of low-grade or transformed non-Hodgkin lymphoma with iodine I-131 tositumomab has good responses.^[57]^ The 4th article is Osmani, Lais et al^[58]^ review of World Health Organization’s classification of non-small cell lung-cancer published in 2018. They think accurate subclassification is very important. Biomarkers is necessary to determine the subclassification of lung-cancer patients in the process of targeted therapies and immunotherapy or monitoring the response of the therapies. Byun, David J. et al^[59]^ discussed in their review about the clinical management of the endocrinopathies including thyroid dysfunctions base on the mechanisms of immune-related adverse events. The research of Ances, BM et al^[60]^ was based on Brain MRI and [F-18] fluorodeoxyglucose - PET assisted imaging suggested several antigens were strongly expressed in the hippocampus neuropil, and the immune marker pattern is sufficient to prompt the diagnosis and predict the treatment response of relate diseases. Another system review written by Abdel-Wahab, Noha et al^[61]^ is about cancer treatment by using antibodies like immune checkpoint blockade adverse effect of immunotherapy. They found out that these types of immunotherapies can lead to autoimmune systemic diseases such as autoimmune colitis, hepatitis, lupus, celiac disease, endocrinopathy, cutaneous, sarcoidosis, polyarthritis, polymyalgia rheumatica, dermatomyositis. Corsello, Salvatore Maria et al^[62]^ introduced the endocrine adverse effects, such as hypophysitis, abnormal thyroid function caused by immunotherapy in their review. However, research on the mechanism and epidemiological investigation of these side effects are not enough. Loibl, S.; Untch, M. et al^[63]^ randomized phase-II study showed that pathological complete response can be increased by the application of durvalumab to anthracycline-/taxane-based neoadjuvant chemotherapy. Goldenberg, DM et al^[64]^ have obtained positive clinical efficacy in the treatment of malignant thyroid with tumor expressing carcinoembryonic antigen by using bispecific antibodies with hapten-radionuclides.

**Table 5:** Top ten Cited papers on Immunotherapy for Thyroid Cancer.

Ranking	Title	Authors	Journals	IF*	Publication year	Total Citations	Average per year
1	Association of tumor mutational burden with outcomes in patients with advanced solid tumors treated with pembrolizumab: prospective biomarker analysis of the multicohort, open-label, phase 2 KEYNOTE-158 study^[51]^	Marabelle, Aurelien et al	*Lancet Oncology*	54.433	2020	698	174.5
2	Safety profiles of anti-CTLA-4 and anti-PD-1 antibodies alone and in combination^[52]^	Boutros, Celine et al	*Nature Reviews Clinical Oncology*	65.011	2016	613	76.63
3	Radioimmunotherapy with iodine I-131 tositumomab for relapsed or refractory B-cell non-Hodgkin lymphoma: updated results and long-term follow-up of the University of Michigan experience^[53]^	Kaminski, MS et al	*Blood*	25.476	2000	409	17.04
4	Current WHO guidelines and the critical role of immunohistochemical markers in the subclassification of non-small cell lung carcinoma (NSCLC): Moving from targeted therapy to immunotherapy^[54]^	Osmani, Lais et al	*Seminars in Cancer Biology*	17.012	2018	396	66
5	Cancer-immunotherapy - immune checkpoint blockade and associated endocrinopathies^[55]^	Byun, David J. et al	*Nature Reviews Endocrinology*	47.564	2017	382	54.57
6	Treatment-responsive limbic encephalitis identified by neuropil antibodies: MRI and PET correlates^[34]^	Ances, BM et al	*Brain*	15.255	2005	330	17.37
7	Adverse events associated with immune checkpoint blockade in patients with cancer: A systematic review of case reports^[56]^	Abdel-Wahab, Noha et al	*Plos One*	3.752	2016	287	35.88
8	Endocrine side effects induced by immune checkpoint inhibitors^[57]^	Corsello, Salvatore Maria et al	*Journal of Clinical Endocrinology & Metabolism*	6.134	2013	285	25.91
9	A randomized phase-II study investigating durvalumab in addition to an anthracycline taxane-based neoadjuvant therapy in early triple-negative breast-cancer: clinical results and biomarker analysis of GeparNuevo study^[58]^	Loibl, S.; Untch, M. et al	*Annals of Oncology*	51.769	2019	275	55
10	Antibody pretargeting advances cancer radioimmunodetection and radioimmunotherapy^[59]^	Goldenberg, DM et al	*Journal of Clinical Oncology*	50.717	2006	267	14.83

IF = impactor factor.

* IF according to Journal Citation Reports (2021).

## 4. Discussion

TC is a very common endocrine tumor. More than 90% of TC has a good prognosis. Recently, the 5-year survival rate reached 98%.^[65]^ The cause of TC is not clear. It says that environmental pollution can cause thyroid disease.^[66]^ Most TC were discovered by ultrasonography, computed tomography, magnetic resonance imaging and fine needle aspiration cytology.^[6,10]^ Artificial intelligence is also for imaging diagnosis of TC.^[67]^ With the increase of TC screening, the morbidity of TC once increased.^[1,68]^ However, due to the change in the scope of screening, the morbidity of TC is on the decline.^[69]^ The morbidity of TC has a certain relationship with countries or regions, and some regions have higher morbidity of TC.^[70]^ In recent years, immunotherapy has been extensively used in cancer treatment including TC.^[26,71–81]^ In this study, papers on immunotherapy for TC were studied by using bibliometric method.

From 1983, relevant research papers began to appear (Fig. 2). In the first 3 decades, the publication of relevant research papers showed a relatively stable period of sluggish growth. The number of papers published during this period accounts for 1 third of the total number of papers. After that, it began to show a rapid growth process, from 13 papers in 2013 to 157 papers in 2022, an increase of more than 12 times.

From the perspective of national or regional output, the top 5 countries or regions are USA, China, France, Italy, Germany. Output of USA papers exceeds twice that of China. In terms of national and regional cooperation, there are 10 cooperation teams (Fig. 3). The highest 5 cooperation teams are the Europe-America cooperation team composed of Italy, Netherlands and Canada. The second is France, Spain, Australia, Belgium and Brazil constituted the Europe-Oceania-South America cooperation team. The third cooperation team includes USA, Romania and Argentina; the 4th includes Switzerland, Poland, Portugal, Singapore. The fifth includes Japan, India and Iran. In terms of the importance of members in the cooperation team, USA has the highest centrality, which indicates that USA is in the most prominent position in the cooperation relationship. From the perspective of immunotherapy for TC, Sweden, USA, Germany, Austria and France began to pay attention to this research earlier. Sweden attention went on a long time, with a span of more than 20 years from 1995 to 2015. China started to pay more attention to this field late, from 2021 to 2022, but it is burst strength is 29.66. This shows that China’s attention to immunotherapy for TC has increased rapidly in the past 2 years.

Of the 1283 institutions that published 963 papers, the top 10 institutions make up about 1, 5th of the total number of papers. Top 10 institutions are mainly from the United States, France, China and Italy. Mayo Clin and Univ Texas MD Anderson Canc Ctr have greater academic influence. INSERM and Memorial Sloan Kettering Cancer Center played a major role in team cooperation.

A total of 5280 authors participated in the creation of all papers. Top 99 authors who have published papers equal to or greater than 2 constitute 6 cooperative teams. The strength of cooperation relationship within and between teams is quite weak. This is shown that the large-scale author cooperation team in the field of immunotherapy for TC is relatively immature.

A total of 384 journals included all the papers. *Journal of Nuclear Medicine* has the greatest papers published (41 papers in total). *Journal of Nuclear Medicine* holds the highest H-Index (25) and G-Index (41). *Clinical Cancer Research* has the highest IF 11.082. The average IF of the top 10 journals is 7.2058. This shows that the field has received considerable attention. In terms of SCI subject category, The *Journal of Nuclear Medicine belongs to Radiology, Nuclear Medicine and Medical Imaging* category; 4 of them are in *Endocrinology and Metabolism* category; 3 of them are in *Oncology* category. From the position of journals in SCI subject category, most journals rank high in SCI category. Two journals rank in the top 10% (2.94%, 6.94%) of their categories; 5 in the highest 25%; 2 ranking around 30%; this shows that the research field is relatively influential in the industry.

In addition to immunotherapy and thyroid cancer, other top keywords are: radioimmunotherapy, immune checkpoint inhibitors, cancer, nivolumab, anaplastic thyroid cancer, prognosis. From immunotherapy, radioimmunotherapy, and thyroid cancer started more than thirty years ago to recent keywords, the molecular imaging, anaplastic thyroid cancer, anaplastic thyroid carcinoma, checkpoint inhibitor, pd-1 (programmeddeath-1), cabozantinib, chemotherapy, immune checkpoint inhibitors, hypothyroidism, medullary-thyroid cancer, we can see the evolution of the research direction (Fig. 6B). The research hotspots in the last 5 years include t-cells, carcinoma, expression, cells, adverse events, survival, management, nivolumab, pembrolizumab, blockade, association guidelines, acquired-resistance, dabrafenib (Fig. 6C). Five large clusters were obtained by clustering analysis of keywords. The largest cluster includes therapy, radioimmunotherapy, medullary-thyroid carcinoma, non hodgkins-lymphoma, monoclonal antibody, antibody, bispecific antibody, monoclonal antibodies, positron-emission-tomography, diagnosis, carcinoembryonic antigen, dosimetry, medullary-thyroid cancer, radiotherapy, bivalent hapten, metastases, thyroid cancer, trial, phase-i trial, radioiodine therapy, receptor radionuclide therapy. The main members of the second cluster are: immunotherapy, cells, t-cells, lung-cancer, antigen, regulatory t-cells, lymphocytes, dendritic cells, prostate-cancer, antitumor immunity, in vitro, tumor, hypothyroidism, in vivo, melanoma patients, thyroid-dysfunction. The third cluster includes nivolumab, adverse events, management, ipilimumab, pembrolizumab, metastatic melanoma, melanoma, open-label, chemotherapy, blockade, association, advanced melanoma, antibodies, cell lung-cancer, cancer-immunotherapy, pd-1, safety, receptor, ipilimumab-induced hypophysitis, squamous-cell carcinoma, docetaxel. The 4th cluster includes carcinoma, phase-ii trial, efficacy, double-blind, phase-ii, prognostic-factors, association guidelines, braf, combination, multicenter, mutations, radioactive iodine. The fifth cluster includes cancer, expression, survival, breast-cancer, tumors, disease, papillary, gene-expression, pd-l1, risk, tumor-associated macrophages, guidelines, autoimmunity. When a keyword gets more attention in a period of time, we call it burst keyword.^[55]^ shows the top 25 keywords with the strongest bursts. The earliest burst keyword was the activated killer cell. The top 3 keywords with high burst strength are: radioimmunotherapy (14.85), monoclonal antibody (13.78), non hodgkins lymphoma (12.54). This shows that these 3 keywords are compelling research hotspots in the corresponding time period. The research hotspots in the past 2 decades include bivalent hapten, dendritic cell, positron-emission tomography, regulatory t cell, metastatic melanoma, ipilimumab, advanced melanoma, adverse event, immune checkpoint inhibitor, management, papillary, blockade, nivolumab (Fig. 7).

The average citations per paper was 28.03. The h-index of the papers was 82. Six of the top 10 articles were published after 2015 (Table 5), indicating that the highly cited articles in this field are quite new. These highly cited literatures have confirmed the efficacy of immunotherapy, and also discussed the side effects or adverse effects of immunotherapy.^[56,59,61,62,64]^ The mechanism and safety of immunotherapy are the main topics that scholars pay heed to.^[57]^ As well as biomarkers for the response to immunotherapy are discussed.^[34,58,63]^ Some scholars discovered that the combination of lenvatinib and pembrolizumab has a long-term positive effect on Anaplastic thyroid carcinoma and metastatic poorly differentiated thyroid carcinomas,^[82]^ Dabrafenib plus trametinib has a good effect for anaplastic TC.^[83]^

This study is a bibliometric analysis based on mathematical and statistical methods to analyze the literature; it has its limitations. Although the WoSCC is recognized as the authoritative scientific literature database, only using the WoSCC data may lead to some data omission. If other database such as Google Scholar and other databases can be included, the analysis results may be more comprehensive. In terms of data standardization, it is difficult to take care of all data because of the large amount of data. For example, the different writing methods of the same institution led to inaccurate data, such as “*Ctr Mol Med and Immunol*” and “*Ctr Molec Med and Immunol*” are processed as 2 different institutions. These could result in bias in the analysis results. However, as the first bibliometric study on immunotherapy for TC, it analyzes the development trend and hotspots of this research field and can provide reference for researchers in this field.

## 5. Conclusion

Holistically, a large number of studies have emerged on immunotherapy for TC, especially in the last decade. USA is in a leading position in this research field from the perspective of scientific research output and cooperation. China, which ranks second in the number of published articles, has performed well in this research field in the past 2 years. Among the institutions, the Garden State Cancer Center has the largest number of papers published. Mayo Clinic and University of Texas MD Anderson Cancer Center have greater academic influence. *Journal of Nuclear Medicine* is the most prolific journal. The research hotspots in the last 5 years include t-cells, carcinoma, expression, cells, adverse events, survival, management, nivolumab, pembrolizumab, blockade, association guidelines, acquired-resistance, dabrafenib. These research hotspots will continue for a while.

In summary, this is the first study on immunotherapy for TC in the way of knowledge map and visualization through bibliometrics. Compared with the conventional reviews, this study uses the research method of bibliometrics to analyze this field. This study provides a valuable reference for future research in the field of immunotherapy for TC.

## Author contributions

**Conceptualization:** Ran Ding, Hongguan Jiao.

**Data curation:** Ran Ding, Hongguan Jiao.

**Formal analysis:** Ran Ding.

**Funding acquisition:** Hongguan Jiao, Weiyi Tian.

**Investigation:** Yuanlin Piao.

**Methodology:** Ran Ding, Hongguan Jiao.

**Project administration:** Hongguan Jiao, Weiyi Tian.

**Resources:** Hongguan Jiao, Yuanlin Piao.

**Software:** Hongguan Jiao.

**Supervision:** Hongguan Jiao, Yuanlin Piao, Weiyi Tian.

**Validation:** Weiyi Tian.

**Visualization:** Yuanlin Piao.

**Writing – original draft:** Ran Ding, Hongguan Jiao.

**Writing – review & editing:** Hongguan Jiao, Yuanlin Piao, Weiyi Tian.

## References

[1] Seib CD Sosa JA. Evolving understanding of the epidemiology of thyroid cancer. Endocrinol Metab Clin North Am. 2019;48:23–35. 30717905 10.1016/j.ecl.2018.10.002

[2] Davies L Welch HG. Current thyroid cancer trends in the United States. JAMA Otolaryngol Head Neck Surg. 2014;140:317–22. 24557566 10.1001/jamaoto.2014.1

[3] Huang JJ Ngai CH Deng YY. Incidence and mortality of thyroid cancer in 50 countries: a joinpoint regression analysis of global trends. Endocrine. 2023;80:355–65. 36607509 10.1007/s12020-022-03274-7

[4] Li YK Huang YQ He X. The global burden of thyroid cancer in high-income Asia-Pacific: a systematic analysis of the global burden of disease study. Ther Adv Endocrinol Metab. 2022;13:12. 10.1177/20420188221090012 PMC9019321 35464880

[5] Vaccarella S Lortet-Tieulent J Colombet M. Global patterns and trends in incidence and mortality of thyroid cancer in children and adolescents: a population-based study. Lancet Diabetes Endocrinol. 2021;9:144–52. 33482107 10.1016/S2213-8587(20)30401-0

[6] Ezzat S Sarti DA Cain DR. Thyroid incidentalomas. Prevalence by palpation and ultrasonography. Arch Intern Med. 1994;154:1838–40. 8053752 10.1001/archinte.154.16.1838

[7] Clerc J. Radioiodine therapy of thyroid autonomy. Q J Nucl Med Mol Imag. 2021;65:138–56. 10.23736/S1824-4785.21.03340-9 33565845

[8] Chun S Lee YS Yu J. Thyroid imaging study in children with suspected thyroid dysgenesis. Ann Pediatr Endocrinol Metab. 2021;26:53–9. 33819958 10.6065/apem.2040120.060 PMC8026338

[9] Janus D Wojcik M Taczanowska A. Follow-up of parenchymal changes in the thyroid gland with diffuse autoimmune thyroiditis in children prior to the development of papillary thyroid carcinoma. J Endocrinol Invest. 2019;42:261–70. 29872995 10.1007/s40618-018-0909-x PMC6394764

[10] Filetti S Durante C Hartl D. Thyroid cancer: ESMO clinical practice guidelines for diagnosis, treatment and follow-updagger. Ann Oncol. 2019;30:1856–83. 31549998 10.1093/annonc/mdz400

[11] Wang WR Chang JT Jia BS. The blood biomarkers of thyroid cancer. Cancer Manag Res. 2020;12:5431–8. 32753960 10.2147/CMAR.S261170 PMC7351621

[12] Porter A Wong DJ. Perspectives on the treatment of advanced thyroid cancer: approved therapies, resistance mechanisms, and future directions. Front Oncol. 2020;10:592202. 33569345 10.3389/fonc.2020.592202 PMC7868523

[13] Gottlieb JA Hill CS Jr. Chemotherapy of thyroid cancer with adriamycin. Experience with 30 patients. N Engl J Med. 1974;290:193–7. 4808917 10.1056/NEJM197401242900404

[14] Xing M. Molecular pathogenesis and mechanisms of thyroid cancer. Nat Rev Cancer. 2013;13:184–99. 23429735 10.1038/nrc3431 PMC3791171

[15] Hanci D Sahin E Muluk NB. Immunotherapy in all aspects. Eur Arch Otorhinolaryngol. 2016;273:1347–55. 25673026 10.1007/s00405-015-3553-5

[16] Nelson HS. 2020 updated asthma guidelines: allergen immunotherapy. J Allergy Clin Immunol. 2020;146:1286–7. 33280713 10.1016/j.jaci.2020.10.011

[17] Dhamija Y Epstein TEG Bernstein DI. Systemic allergic reactions and anaphylaxis associated with allergen immunotherapy. Immunol Allergy Clin North Am. 2022;42:105–19. 34823741 10.1016/j.iac.2021.09.012

[18] Wright JJ Powers AC Johnson DB. Endocrine toxicities of immune checkpoint inhibitors. Nat Rev Endocrinol. 2021;17:389–99. 33875857 10.1038/s41574-021-00484-3 PMC8769055

[19] Zhang H Zhu JF Fang TX. Supramolecular biomaterials for enhanced cancer immunotherapy. J Mater Chem B. 2022;10:7183–93. 35348177 10.1039/d2tb00048b

[20] Zeng YP Li SF Zhang SF. Cell membrane coated-nanoparticles for cancer immunotherapy. Acta Pharm Sin B. 2022;12:3233–54. 35967284 10.1016/j.apsb.2022.02.023 PMC9366230

[21] Yang M Olaoba OT Zhang CY. Cancer immunotherapy and delivery system: an update. Pharmaceutics. 2022;14:1630. 36015256 10.3390/pharmaceutics14081630 PMC9413869

[22] Xia MQ Wang S Ye YC. Effect of the m6ARNA gene on the prognosis of thyroid cancer, immune infiltration, and promising immunotherapy. Front Immunol. 2022;13:18. 10.3389/fimmu.2022.995645 PMC9664221 36389678

[23] Wu P Shi JY Wang ZY. Evaluate the immune-related eRNA models and signature score to predict the response to immunotherapy in thyroid carcinoma. Cancer Cell Int. 2022;22:18. 36217201 10.1186/s12935-022-02722-8 PMC9549686

[24] Wang GL Miao C Mo LJ. MYCBP2 expression correlated with inflammatory cell infiltration and prognosis immunotherapy in thyroid cancer patients. Front Immunol. 2022;13:12. 10.3389/fimmu.2022.1048503 PMC9792662 36582246

[25] Wachter S Roth S Gercke N. Anti-proliferative effect of combined radiotherapy and immunotherapy in anaplastic thyroid cancer cells. Chirurgie. 2022;93:1183–1183. 10.3390/life13061397 PMC10301015 37374179

[26] Qiu WG Wu XQ Shi HH. ASF1B: a possible prognostic marker, therapeutic target, and predictor of immunotherapy in male thyroid carcinoma. Front Oncol. 2022;12:14. 10.3389/fonc.2022.678025 PMC8841667 35174076

[27] Zyoud SH Al-Jabi SW Amer R. Global research trends on the links between the gut microbiome and cancer: a visualization analysis. J Transl Med. 2022;20:83. 35148757 10.1186/s12967-022-03293-y PMC8832721

[28] Zhong W Shen Z Wu Y. Knowledge mapping and current trends of immunotherapy for prostate cancer: a bibliometric study. Front Immunol. 2022;13:1014981. 36389756 10.3389/fimmu.2022.1014981 PMC9647028

[29] Zhao PY Jiao YN Ma ZF. Publication trends and hotspots of drug resistance in colorectal cancer during 2002-2021: a bibliometric and visualized analysis. Front Oncol. 2022;12:947658. 36110958 10.3389/fonc.2022.947658 PMC9469653

[30] Yang S Zhao S Ye Y. Global research trends on the links between gut microbiota and cancer immunotherapy: a bibliometric analysis (2012-2021). Front Immunol. 2022;13:952546. 36090978 10.3389/fimmu.2022.952546 PMC9449151

[31] Berry D Widder S. Deciphering microbial interactions and detecting keystone species with co-occurrence networks. Front Microbiol. 2014;5:14. 24904535 10.3389/fmicb.2014.00219 PMC4033041

[32] Shen ZF Wu HY Chen ZS. The global research of artificial intelligence on prostate cancer: a 22-year bibliometric analysis. Front Oncol. 2022;12:16. 10.3389/fonc.2022.843735 PMC8921533 35299747

[33] Chen C. CiteSpace II: detecting and visualizing emerging trends and transient patterns in scientific literature. J Am Soc Inf Sci Technol. 2006;57:359–77.

[34] van Eck NJ Waltman L. Software survey: VOSviewer, a computer program for bibliometric mapping. Scientometrics. 2010;84:523–38. 20585380 10.1007/s11192-009-0146-3 PMC2883932

[35] Orduna-Malea E Costas R. Link-based approach to study scientific software usage: the case of VOSviewer. Scientometrics. 2021;126:8153–86.

[36] Hou JH Yang XC Chen CM. Emerging trends and new developments in information science: a document co-citation analysis (2009-2016). Scientometrics. 2018;115:869–92.

[37] Batagelj V Mrvar A. Pajek - analysis and visualization of large networks. In: Mutzel P Junger M Leipert S (eds). Graph drawing. Vol 2265. Berlin: Springer-Verlag Berlin; 2002:477–78.

[38] Zhou J Jiang YJ Pantelous AA. A systematic review of uncertainty theory with the use of scientometrical method. Fuzzy Optim Decis Mak. 2022;22:463–518.

[39] Hassan-Montero Y De-Moya-Anegon F Guerrero-Bote VP. SCImago graphica: a new too for exploring and visually communicating data. Prof Inf. 2022;31:12.

[40] He TM Ao JW Duan CC. Bibliometric and visual analysis of nephrotoxicity research worldwide. Front Pharmacol. 2022;13:15. 10.3389/fphar.2022.940791 PMC9515790 36188597

[41] Thakur RR Singh P. Study of carrier scattering and quantization effects in steep retrograded gate all around FinFETs for nano technology applications. Paper presented at: International Multi-Conference on Computing, Communication, Electrical and Nanotechnology (I2CN); April 25–26, 2019; Ettumanoor, INDIA.

[42] van Eck NJ Waltman L. Citation-based clustering of publications using CitNetExplorer and VOSviewer. Scientometrics. 2017;111:1053–70. 28490825 10.1007/s11192-017-2300-7 PMC5400793

[43] Kleinberg J. Bursty and hierarchical structure in streams. Data Min Knowl Discov. 2003;7:373–97.

[44] Chen C. Searching for intellectual turning points: progressive knowledge domain visualization. Proc Natl Acad Sci U S A. 2004;101:5303–10. 14724295 10.1073/pnas.0307513100 PMC387312

[45] Pan XL Yan EJ Cui M. Examining the usage, citation, and diffusion patterns of bibliometric mapping software: a comparative study of three tools. J Inform. 2018;12:481–93.

[46] Colares GS Dell’Osbel N Wiesel PG. Floating treatment wetlands: a review and bibliometric analysis. Sci Total Environ. 2020;714:136776. 31991269 10.1016/j.scitotenv.2020.136776

[47] Buyukkidik S. A bibliometric analysis: a tutorial for the bibliometrix package in R using IRT literature. J Meas Eval Educ Psychol-EPOD. 2022;13:164–93.

[48] Aria M Cuccurullo C. bibliometrix: an R-tool for comprehensive science mapping analysis. J Inform. 2017;11:959–75.

[49] Legerfo P Feind C Weber C. Immunotherapy of thyroid-cancer by induction of autoimmune-thyroiditis. Surgery. 1983;94:959–65. 6648811

[50] Singh PK. t-Index: entropy based random document and citation analysis using average h-index. Scientometrics. 2022;127:637–60.

[51] Sanyal DK Dey S Das PP. g(m)-index: a new mentorship index for researchers. Scientometrics. 2020;123:71–102.

[52] Robinson DBT Hopkins L Brown C. Relative value of adapted novel bibliometrics in evaluating surgical academic impact and reach. World J Surg. 2019;43:967–72. 30564922 10.1007/s00268-018-04893-w

[53] Thompson DF Callen EC Nahata MC. New indices in scholarship assessment. Am J Pharm Educ. 2009;73:111. 19885080 10.5688/aj7306111 PMC2769533

[54] Schreiber M. Fractionalized counting of publications for the g-index. J Am Soc Inf Sci Technol. 2009;60:2145–50.

[55] Marabelle A Fakih M Lopez J. Association of tumour mutational burden with outcomes in patients with advanced solid tumours treated with pembrolizumab: prospective biomarker analysis of the multicohort, open-label, phase 2 KEYNOTE-158 study. Lancet Oncol. 2020;21:1353–65. 32919526 10.1016/S1470-2045(20)30445-9

[56] Boutros C Tarhini A Routier E. Safety profiles of anti-CTLA-4 and anti-PD-1 antibodies alone and in combination. Nat Rev Clin Oncol. 2016;13:473–86. 27141885 10.1038/nrclinonc.2016.58

[57] Kaminski MS Estes J Zasadny KR. Radioimmunotherapy with iodine I-131 tositumomab for relapsed or refractory B-cell non-Hodgkin lymphoma: updated results and long-term follow-up of the University of Michigan experience. Blood. 2000;96:1259–66. 10942366

[58] Osmani L Askin F Gabrielson E. Current WHO guidelines and the critical role of immunohistochemical markers in the subclassification of non-small cell lung carcinoma (NSCLC): moving from targeted therapy to immunotherapy. Semin Cancer Biol. 2018;52:103–9. 29183778 10.1016/j.semcancer.2017.11.019 PMC5970946

[59] Byun DJ Wolchok JD Rosenberg LM. Cancer immunotherapy - immune checkpoint blockade and associated endocrinopathies. Nat Rev Endocrinol. 2017;13:195–207. 28106152 10.1038/nrendo.2016.205 PMC5629093

[60] Ances BM Vitaliani R Taylor RA. Treatment-responsive limbic encephalitis identified by neuropil antibodies: MRI and PET correlates. Brain. 2005;128:1764–77. 15888538 10.1093/brain/awh526 PMC1939694

[61] Abdel-Wahab N Shah M Suarez-Almazor ME. Adverse events associated with immune checkpoint blockade in patients with cancer: a systematic review of case reports. PLoS One. 2016;11:e0160221. 27472273 10.1371/journal.pone.0160221 PMC4966895

[62] Corsello SM Barnabei A Marchetti P. Endocrine side effects induced by immune checkpoint inhibitors. J Clin Endocrinol Metab. 2013;98:1361–75. 23471977 10.1210/jc.2012-4075

[63] Loibl S Untch M Burchardi N. A randomised phase II study investigating durvalumab in addition to an anthracycline taxane-based neoadjuvant therapy in early triple-negative breast cancer: clinical results and biomarker analysis of GeparNuevo study. Ann Oncol. 2019;30:1279–88. 31095287 10.1093/annonc/mdz158

[64] Goldenberg DM Sharkey RM Paganelli G. Antibody pretargeting advances cancer radioimmunodetection and radioimmunotherapy. J Clin Oncol. 2006;24:823–34. 16380412 10.1200/JCO.2005.03.8471

[65] Miller KD Nogueira L Devasia T. Cancer treatment and survivorship statistics, 2022. CA Cancer J Clin. 2022;72:409–36. 35736631 10.3322/caac.21731

[66] Al Sharabati M Abokwiek R Al-Othman A. Biodegradable polymers and their nano-composites for the removal of endocrine-disrupting chemicals (EDCs) from wastewater: a review. Environ Res. 2021;202:111694. 34274334 10.1016/j.envres.2021.111694

[67] Sorrenti S Dolcetti V Radzina M. Artificial intelligence for thyroid nodule characterization: where are we standing? Cancers (Basel). 2022;14:3357. 35884418 10.3390/cancers14143357 PMC9315681

[68] Schuman AD Spector ME Jaffe CA. Changes in diagnosis of thyroid cancer among medicaid beneficiaries following medicaid expansion. JAMA Surg. 2020;155:1080–1. 32936239 10.1001/jamasurg.2020.3290 PMC7495325

[69] Siegel RL Miller KD Fuchs HE. Cancer statistics, 2022. CA Cancer J Clin. 2022;72:7–33. 35020204 10.3322/caac.21708

[70] Kang MJ Won YJ Lee JJ. Cancer statistics in Korea: incidence, mortality, survival, and prevalence in 2019. Cancer Res Treat. 2022;54:330–44. 35313102 10.4143/crt.2022.128 PMC9016309

[71] Yang FF Hu T Liu JQ. Histone deacetylases (HDACs) as the promising immunotherapeutic targets for hematologic cancer treatment. Eur J Med Chem. 2023;245:13. 10.1016/j.ejmech.2022.114920 36399875

[72] Yuan M Zhai YR Hui ZG. Application basis of combining antiangiogenic therapy with radiotherapy and immunotherapy in cancer treatment. Front Oncol. 2022;12:8. 10.3389/fonc.2022.978608 PMC9681994 36439496

[73] Singh V Sheikh A Abourehab MAS. Dostarlimab as a miracle drug: rising hope against cancer treatment. Biosensors (Basel). 2022;12:617. 36005013 10.3390/bios12080617 PMC9406030

[74] Sanatkar SA Heidari A Rezaei N. Cancer immunotherapy: diverse approaches and obstacles. Curr Pharm Des. 2022;28:2387–403. 35909273 10.2174/1381612828666220728160519

[75] Rostamizadeh L Molavi O Rashid M. Recent advances in cancer immunotherapy: modulation of tumor microenvironment by Toll-like receptor ligands. Bioimpacts. 2022;12:261–90. 35677663 10.34172/bi.2022.23896 PMC9124882

[76] Zhong X Xu S Wang QH. CAPN8 involves with exhausted, inflamed, and desert immune microenvironment to influence the metastasis of thyroid cancer. Front Immunol. 2022;13:14. 10.3389/fimmu.2022.1013049 PMC9647051 36389799

[77] Zhang PT Guan HX Yuan SK. Targeting myeloid derived suppressor cells reverts immune suppression and sensitizes BRAF-mutant papillary thyroid cancer to MAPK inhibitors. Nat Commun. 2022;13:18. 35332119 10.1038/s41467-022-29000-5 PMC8948260

[78] Xie WJ Zeng Y Hu LF. Based on different immune responses under the glucose metabolizing type of papillary thyroid cancer and the response to anti-PD-1 therapy. Front Immunol. 2022;13:15. 10.3389/fimmu.2022.991656 PMC9536150 36211409

[79] Tarabichi M Demetter P Craciun L. Thyroid cancer under the scope of emerging technologies. Mol Cell Endocrinol. 2022;541:14. 10.1016/j.mce.2021.111491 34740746

[80] Shih SR Chen KH Lin KY. Immunotherapy in anaplastic thyroid cancer: case series. J Formos Med Assoc. 2022;121:1167–73. 35031200 10.1016/j.jfma.2022.01.003

[81] Ragusa F Ferrari SM Elia G. Combination strategies involving immune checkpoint inhibitors and tyrosine kinase or BRAF inhibitors in aggressive thyroid cancer. Int J Mol Sci. 2022;23:5731. 35628540 10.3390/ijms23105731 PMC9144613

[82] Dierks C Seufert J Aumann K. Combination of lenvatinib and pembrolizumab is an effective treatment option for anaplastic and poorly differentiated thyroid carcinoma. Thyroid. 2021;31:1076–85. 33509020 10.1089/thy.2020.0322 PMC8290324

[83] Subbiah V Kreitman RJ Wainberg ZA. Dabrafenib plus trametinib in patients with BRAF V600E-mutant anaplastic thyroid cancer: updated analysis from the phase II ROAR basket study. Ann Oncol. 2022;33:406–15. 35026411 10.1016/j.annonc.2021.12.014 PMC9338780

